# Checkpoint blockade therapy for functioning pituitary adenomas

**DOI:** 10.18632/oncoscience.509

**Published:** 2020-06-01

**Authors:** Selena J. Lorrey, Hanna R. Kemeny, Peter E. Fecci

**Affiliations:** ^1^Department of Immunology, Duke University Graduate School, Durham, NC, USA; ^2^Brain Tumor Immunotherapy Program, Duke University Medical Center, Durham, NC, USA; ^3^Department of Neurosurgery, Northwestern University, Chicago, IL, USA; ^4^Department of Neurosurgery, Duke University School of Medicine, Durham, NC, USA

**Keywords:** checkpoint blockade, immunotherapy, pituitary adenoma, CTLA-4, PD-1, PD-L1, ACTH, cushing's disease, hypophysitis; brain tumor

In recent years, immunotherapy has become an increasingly frontline treatment for many solid tumors. Checkpoint blockade, in particular, has achieved significant success across a variety of histologies [1], and has typically taken the form of antibodies targeting CTLA-4 and/or the PD-1/PD-L1 axis. Such therapies are currently FDA-approved for use against numerous more immunogenic cancers. Our group, however, recently published data in the November 2019 issue of *Clinical Cancer Research* that checkpoint blockade (anti-PD-L1) may also be particularly well-suited to functioning pituitary adenomas, such as those causing Cushing’s disease (CD) [2]. While these tumors are technically benign, their clinical course is not, and there remains a tremendous clinical need for therapies that can prevent the significant morbidity and mortality that result from CD. The specific utility of checkpoint blockade against these tumors was suggested by two key findings: 1) the classic side effect of hypophysitis in patients treated with immune checkpoint blockade for other cancers (implying the capacity for these therapies to elicit a T cell response in the gland) [3]; and 2) the discovery of increased levels of PD-L1 on the surface of functioning adenomas [4].

CD is a condition resulting from hypersecretion of adrenocorticotropic hormone (ACTH) by a pituitary adenoma, resulting in excessive levels of cortisol production by the adrenal glands. Due to this metabolic cascade, patients can experience a number of clinical sequalae including excessive weight gain, metabolic abnormalities (such as diabetes), immunodeficiency, and a variety of other complications [5]. There is a significant mortality rate from these sequelae. This niche patient population is currently underserved by standard-of care therapeutic options. While resection of the tumor remains the first-line treatment for CD patients, approximately 25% will recur within 5 years of an initial surgical resection [6]. There is a dearth of effective treatment options upon recurrence, therefore applying checkpoint blockade therapy to patients with refractory CD becomes an exciting prospect.

Further detailing our rationale, previous studies found that advanced melanoma or prostate cancer patients treated with ipilimumab (anti-CTLA4 monoclonal antibody), experienced inflammation of the pituitary gland, known as secondary hypophysitis. This inflammation was deemed on-target, off-tumor toxicity, as normal pituitary glands were subsequently shown to express CTLA-4 [3]. The capacity, then, for checkpoint blockade to elicit an immune response within the intracranially located pituitary gland was previously demonstrated. Likewise, pituitary adenomas themselves exhibit T cell infiltrates, a pre-requisite for checkpoint blockade efficacy [4]. The case for testing anti-PD-L1, specifically, was made when functioning pituitary adenomas (those producing hormones) were found to upregulate surface levels of PD-L1 [4].

Through our work, we showed that human ACTH-secreting pituitary adenomas exhibit both PD-L1 expression and T cell infiltration, and that these findings could be recapitulated in a novel murine model of CD. We found that treatment with anti-PD-L1 restricts tumor growth both in subcutaneous and intracranial models. Additionally, we demonstrated that anti-PD-L1 treatment reduced ACTH production, the main harbinger of morbidity from CD [2].

These encouraging data suggest that patients with refractory CD may indeed benefit from treatment with checkpoint blockade therapy. Accordingly, there is a single case report of a patient with recurrent ACTH-producing pituitary carcinoma who was treated with a combination of ipilimumab (anti-CTLA-4) and nivolumab (anti-PD-1). This combination therapy effectively reduced ACTH levels from greater than 45,000pg/mL to 66pg/mL and resulted in regression of both intracranial and peripheral tumors [7]. The prospect of redirecting checkpoint blockade therapy for use in pituitary adenomas thus seems especially promising. Likewise, while we have tested anti-PD-L1 therapies, the expression of CTLA-4 on the normal gland, combined with the case report results above, certainly suggest that anti-CTLA-4 may be an additional, cogent treatment option.
